# *Enterococcus faecium B13* Affects Mice Growth by Regulating Gut Microbiota and Metabolites

**DOI:** 10.1002/fsn3.71192

**Published:** 2025-11-28

**Authors:** Fan Luo, Jiajun Wu, Shumin Wang, Ming Zhang, Yixuan Song, Yaqiu Lin

**Affiliations:** ^1^ College of Animal and Veterinary Sciences Southwest Minzu University Chengdu China

**Keywords:** differential metabolites, *Enterococcus faecium*, growth performance, gut microbiota

## Abstract

Gut microbiota can influence the growth performance of animals. In our previous study, *Enterococcus faecium B13* exhibited promoting growth in piglets. This study aimed to reveal the mechanism underlying the growth promotion. Therefore, B13 was administered to male mice via oral gavage, and the growth performance, gut microbiota, and metabolites were analyzed. Results showed that the weight gain trend in the B13 group was slightly higher than in the control group during 28 days, and the serum triglycerides (TG) level was significantly lower in the B13 group (*p* < 0.05). Notably, the microbial composition differed between the two groups. The ratio of *Firmicutes/Bacteroidetes* (F/B) rose in the colon. The *Allobaculum* and *Alloprevotella,* which promoted growth and inhibited obesity, were significantly different genera in the ileum and colon, separately. On the 11th day after B13 withdrawal, all differential genera, except for *Alloprevotella* and *RF39*, returned to normal levels. A total of 53 differential metabolites were identified by Liquid Chromatograph Mass Spectrometer (LC–MS), with the most significantly altered substances mainly involved in the arachidonic acid metabolic pathway, arginine and ornithine metabolic pathway, arginine and proline metabolic pathway and linoleic acid metabolic pathway. Additionally, Spearman's correlation analysis indicated a significant correlation between the *Alloprevotella* and the main differential metabolites. All these results suggest that *Alloprevotella* might play a major role under the conditions of this experiment. This study revealed the possible growth‐promoting mechanism of B13 and suggests its potential as a probiotic.

## Graphical abstract

This study showed that *E. faecium B13* could potentially improve growth performance by modulating the gut microbiota and metabolites, of which *Alloprevotella* and arginine perhaps were the main targets regulated by B13. Spearman's correlation analysis revealed that *Alloprevotella* was related to the fatty acid belonging to the arachidonic acid metabolic and linoleic acid metabolic pathway.

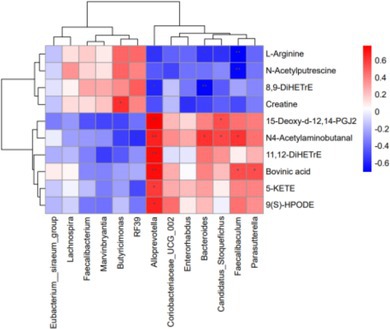

## 1 Introduction

In recent years, an increasing mass of data has demonstrated that probiotic microorganism supplements can effectively intervene in animal health. Probiotic microbes play an important role in enhancing absorption and digestion in the gut, improving production performance and feed digestibility, preventing, and treating diarrhea and stresses, strengthening host immunity, reducing metabolic disorders, etc. (Ding et al. 2021; Liao and Nyachoti 2017; Liu et al. 2018). Lactic acid bacteria (LAB) as the main probiotics, have been used both in healthy and animal nutrition. Many *Lactobacillus* and *Lactococcus*, like *Lactobacillus reuteri* , *Lactobacillus rhamnosus GG*, *Leuconostocpseudomesenteroides*, and so on, are approved as feed, edible, and even pharmaceutical probiotics by the government departments of different countries (Garcia et al. 2020; Koutsoumanis et al. 2020).

*Enterococcus faecium* , a kind of LAB, is highly controversial for now because some strains have emerged to become significant nosocomial pathogens, acquiring extensive antibiotic resistance. But some of them, for example, *E. faecium EF1*, *E. faecium NCIMB 11181*, *E. faecium SF68*, and *E. faecium M‐74*, are beneficial and often used as commercial probiotics (Shao et al. 2022). For example, *E. faecium SF68* is a well‐known probiotic with a long history of safe use, which has been authorized for use as a probiotic in pharmaceutical preparations and food supplements in humans and animals (Holzapfel et al. 2018; Liu et al. 2023; Greuter et al. 2020). *E. faecium EF1* was proved to be associated with a remarkable increase in the body weight of piglets for both suckling and weaning periods and also decreased the diarrhea rate (Park et al. 2024). *E. faecium* could resist low pH, bile salts, and high temperatures, and encountered digestion, so they could adhere in the intestine to function (Zommiti et al. 2018; Hanchi et al. 2018; Zhang et al. 2024).

Now, probiotic *E. faecium* strains are widely used in the production of poultry, swine, and aquaculture species. Numerous studies (Du et al. 2021; Sureshkumar et al. 2022; Martin et al. 2025) have demonstrated that feeding *E. faecium* can improve animal growth performance, including reducing the feed conversion ratio (FCR) and increasing average daily gain (ADG) and body weight (BW) and so on. These changes in performance are often accompanied by alterations in the gut microbiota, yet there are no further in‐depth reports on the characteristics of microbial community changes or key microorganisms to drive the improvement of growth performance.

Previous research has shown that *E. faecium* B13 was a probiotic (Xiao et al. 2024) and feeding B13 can increase daily weight gain of piglets (Ding Shuang et al. 2017), but the mechanism is unclear. This study aims to explore the potential interaction network relationship between B13, gut core microbiota, and gut metabolites through combined analysis of microbiome and metabolome, aiming to reveal the mechanism of action of B13.

## 2 Materials and Methods

### 2.1 Strain and Culture Condition

*E. faecium B13* isolated from fermented pickled chili was evaluated for its safety and probiotic properties (Xiao et al. 2024), and preserved in China General Microbiological Culture Collection Center (CGMCC, Beijing) with preservation number 23693. It was cultured in DeMan‐Rogosa‐Sharpe (MRS) medium for 24 h at 30°C; then the bacterial broth was centrifuged at 6000 rpm for 15 min and the sediment was resuspended in sterile saline (0.9%), adjusting the concentration to 10^8^ CFU/mL.

### 2.2 Animal Experiment Design

Three‐week‐old ICR male mice were housed under room temperature conditions of 25°C ± 1°C, air humidity of 60% ± 10%, and a 12/12 h diurnal cycle. All ICR mice had free access to the basal diet and water. After acclimatization for 1 week, male mice were randomly divided into two batches, with each batch divided into two groups: control group with 200 μL of sterile saline and B13 group with 200 μL B13 suspension (1 × 10^8^ CFU/mL). Weight was regularly measured, and padding was replaced every 3 days. Mice were monitored for general health, feeding and residuals were accurately recorded daily. After 28 days, one batch of mice was randomly selected from each group (*n* = 8) and euthanized to determine relevant indicators (control group = 8, B13 group = 6). The remaining mice were stopped gavage and maintained on the normal diet until the 11th day. At the end of each experiment all mice (control group = 7, B13 group = 7) were fasted for 12 h and euthanized, and animal tissues were collected as below for characterization. The basal diet was purchased from Chengdu Dossy Experimental Animals Co. Ltd. (Chengdu, China) (Table 1).

**TABLE 1:** The compositions and nutrient levels of the basal diets (air‐dry basis, %).

Ingredients	Content	Nutrient levels	Content
Corn	27.70	Water	18.8
Wheat	30.60	Protein	37
Soybean	12.00	Fat	10.4
Soybean meal	4.00	Fiber	7
Rice bran	6.00	Carbohydrate	9.62
Alfalfa meal	10.00	Ash	13.2
Fish meal	6.00	Calcium	2.26
Premix	3.70	Phosphorus	1.72

### 2.3 Sample Collection

At the end of the feeding experiment, ICR mice were euthanized, and the hearts, livers, spleens, and kidneys were weighed accurately. The ileal and colonic contents were collected aseptically and stored at −80°C. On the 11th day after stopping feeding B13, the colonic contents were collected using the same methods.

### 2.4 Serum Biochemistry Profiles

Blood from mouse heart was centrifuged at 3000 r/min for 15 min to obtain serum, and the levels of triglycerides (TG), total cholesterol (TC), and blood glucose (GIU) in the serum were detected using commercially available kits (Nanjing Jiancheng Bioengineering Institute, Jiangsu, China), according to the manufacturer's instructions.

### 2.5 Microbiota Profiling in Ileum and Colon

Genomic DNA of the ileal and colonic contents was extracted using a DNA extraction kit. (Bio Takara) and tested using agarose gel electrophoresis. The primers 343F (5′‐TACGGRAGGCAGCAG‐3′) and 798R (5′‐AGGGTATCTAATCCT‐3′) were used to amplify the V3–V4 region of the 16SrRNA gene. The library sequencing and data processing were conducted by OE Biotech Co. Ltd. (Shanghai, China). Raw sequencing data were in FASTQ format. Paired‐end reads were then preprocessed using cut adapt software to detect and cut off the adapter. After trimming, paired‐end reads were filtered for low‐quality sequences, denoised, merged and detected and cut off the chimera reads using DADA2 (Lee et al. 2021) with the default parameters of QIIME2 (Bolyen et al. 2019). At last, the software outputs the representative reads and the ASV abundance table. The representative read of each ASV was selected using the QIIME 2 package. All representative reads were annotated and blasted against the Silva database Version 138 using q2‐feature‐classifier with the default parameters.

### 2.6 Metabolome Analysis of the Colonic Content

The metabolites of mice colonic contents were analyzed using a Waters ACQUITY (Waters, USA) UPLC system with an ACQUITY UPLC HSS T3 (2.1 × 150 mm, 1.8 μm) (Waters, Milford, MA, USA). The experimental conditions of LC were set according to the method reported by Acuña et al. (2021). Data were acquired using a Thermo Q Exactive mass spectrometer (Thermo Fisher Scientific, USA) with an electrospray ionization source (ESI) in positive and negative ion modes, and dynamic exclusion was used to remove unnecessary MS/MS information (Liu et al. 2019).

### 2.7 Statistical and Bioinformatics Analysis

All data were expressed as the mean ± standard deviation (SD) of three independent experiments. Experimental data were analyzed by *T*‐test. *p* < 0.05 was considered statistically different and *p* < 0.01 was a highly significant difference.

16S rRNA gene sequencing analysis was performed using the QIIME2 and R package (v3.2.0). The UniFrac distance metric was used to investigate structural changes of microbial communities between samples and the result was visualized by Principal Coordinate Analysis (PCoA). Linear discriminant analysis Effect Size (LEfSe) was performed to detect taxa that were rich in differences between the two groups using default parameters.

LC–MS untargeted metabolomic analysis was performed using the R package Ropls. *p*‐value, variable projection importance (VIP) and fold change (FC) between groups were measured to assist in the screening of marker metabolites. Metabolite molecules were considered statistically significant when the *p* < 0.05, VIP > 1.

Spearman correlation analysis is used to determine the correlation between intestinal bacteria and metabolites.

## 3 Results

### 3.1 Growth Performance and Organ Index of Mice

**FIGURE 1:**
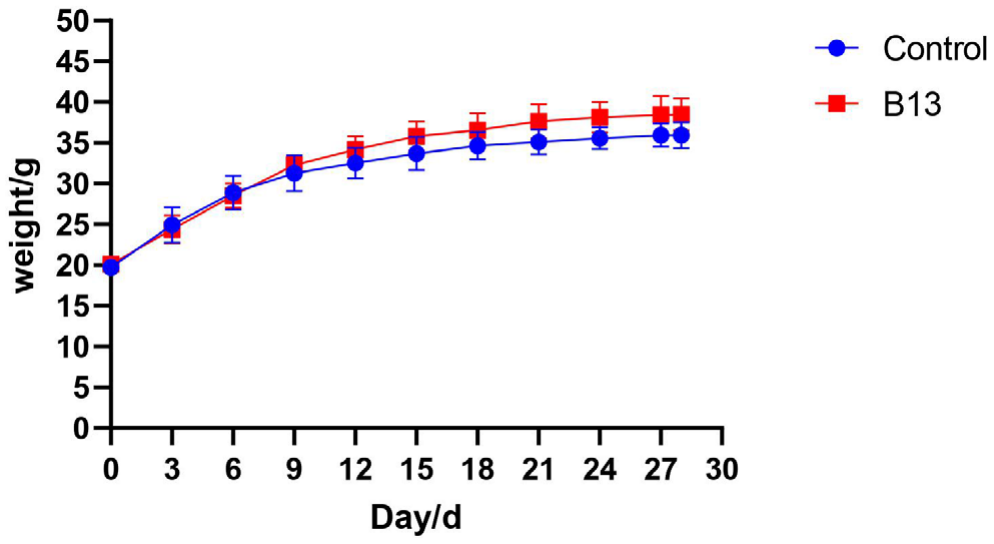
Body weight change curve during 28 days.

During the 4‐week feeding, the final body weight, average daily gain (ADG), average daily feed intake (ADFI), feed‐to‐weight ratio (F/R), and organ index were not significantly different between the two groups (Tables S1 and S2). Body weight of mice in group B13 was slightly higher than that in the control group (Figure 1).

### 3.2 Serum Lipid and Blood Glucose

Compared with the control group, the serum triglycerides (TG) level was significantly lower in the B13 group (*p* < 0.05), while the levels of total cholesterol (TC) and blood glucose (GIU) were not significantly different (Table 2).

**TABLE 2:** Effect of *E. faecium B13* on serum biochemical index in mice.

Items	Control group (*n* = 8)	B13 group (*n* = 6)	*p*
Triglycerides (TG) (mmol/L)	3.52 ± 0.18	2.22 ± 0.50	< 0.01
Total cholesterol (TC) (mmol/L)	20.09 ± 4.13	17.84 ± 4.84	0.388
Blood glucose (GIU) (mmol/L)	8.48 ± 3.77	9.55 ± 1.14	0.519

### 3.3 Microbiota in Ileum

**FIGURE 2:**
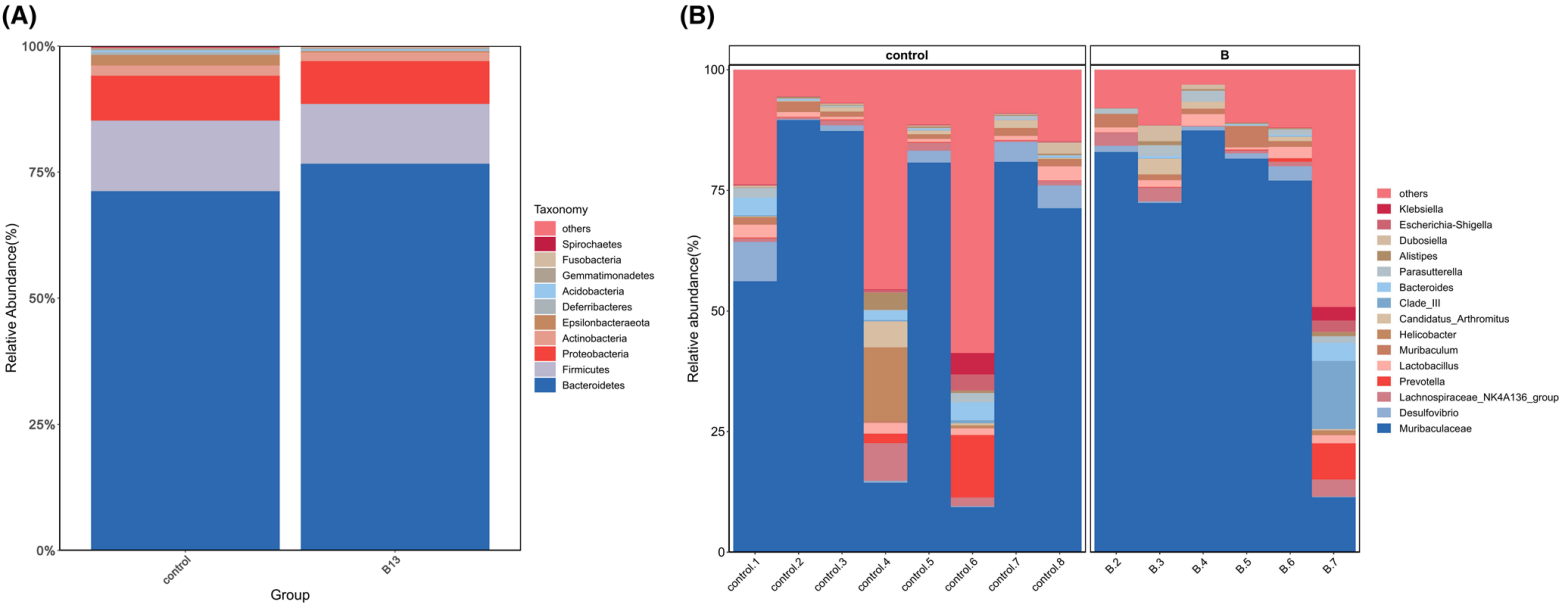
Community composition in ileum at the phylum and genus levels on day 28. (A) Phylum‐level species distribution bar chart, showing only the top 10 species; (B) Genus‐level species distribution bar chart, showing only the top 15 species.

*E. faecium B13* did not significantly affect ileal microbial diversity and abundance and the microbial composition showed no significant difference between the two groups at either the phylum level (Figure 2A) or the genus level (Figure 2B). At the genus level, the relative abundance of *Allobaculum* significantly increased (*p* < 0.05) in group B13, which was the only genus that showed significant differences in the top 30 genera. Moreover, there were 8 differential biomarkers (*p* < 0.05) at the genus level between the two groups (Figure S1). Ying et al. reported that ileal microbiota have a significant impact on the growth performance of broiler chickens; therefore, the role of *Allobaculum* in the ileum requires further investigation (Zhang et al. 2024).

### 3.4 Colonic Microbiota on the 29th Day

There was no significant difference in the bacterial α‐diversity indices (Shannon, Chao 1, Simpson, and ACE indices) between the two groups (*p* > 0.05) (Table 3).

**TABLE 3:** Effect of *E. faecium B13* on microbial α‐diversity analysis in colon.

Item	Control (*n* = 8)	B13 group (*n* = 6)	*p*
Chao 1	422.07 ± 54.87	385.48 ± 65.85	0.738
Shannon	6.62 ± 0.26	6.53 ± 0.39	0.333
Simpson	0.97 ± 0.008	0.98 ± 0.006	0.535
Ace	422.51 ± 55.54	385.41 ± 65.97	0.756

**FIGURE 3:**
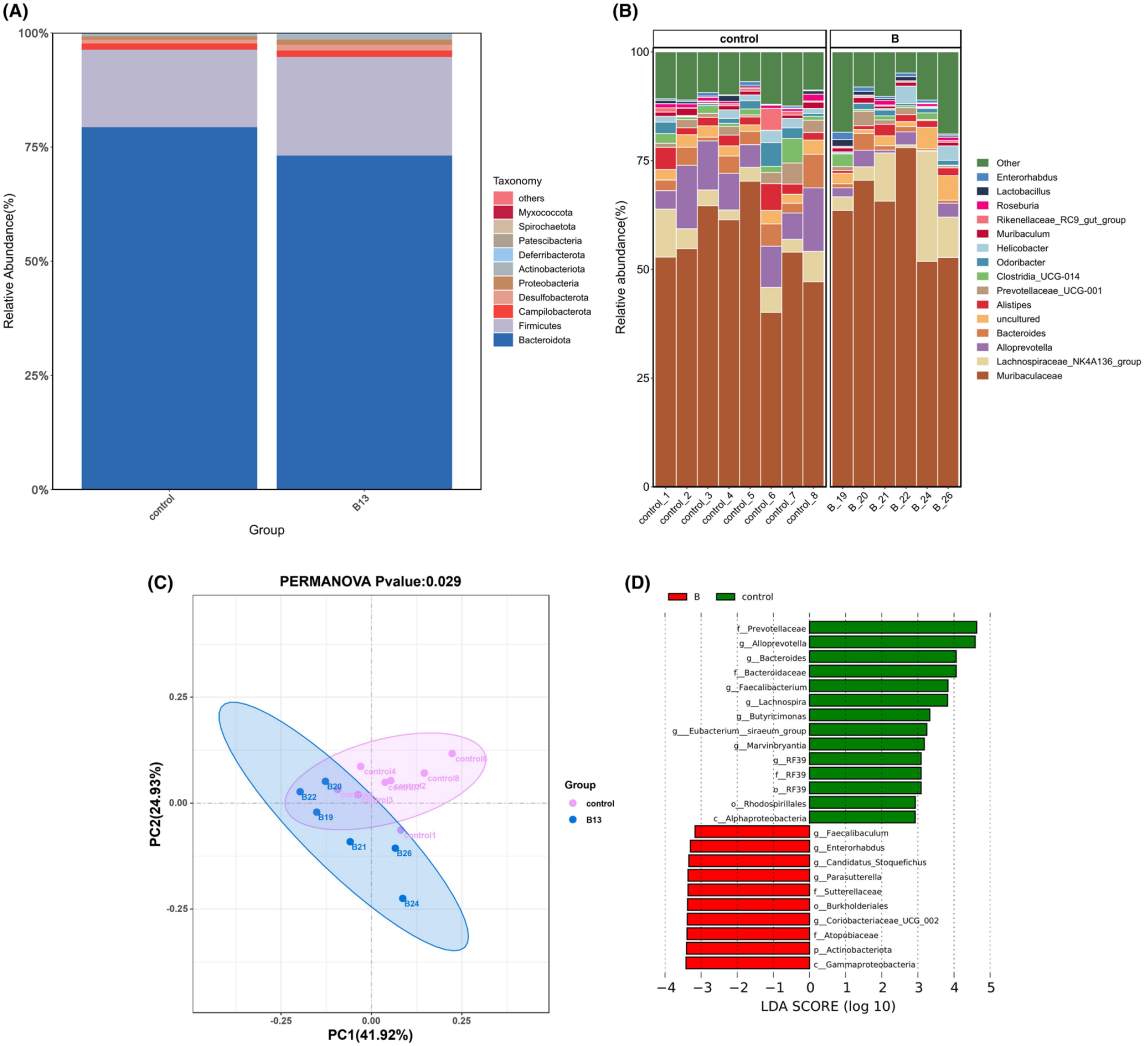
Community composition and differential analysis between groups in colon on day 28. (A) Phylum‐level species distribution bar chart, showing only the top 10 species. (B) Genus‐level species distribution bar chart, showing only the top 15 species. (C) PCoA analysis based on Bray Curtis distance matrix algorithm. (D) LEfSe analysis of the colon microflora, displaying only genera with absolute values of linear discriminant analysis (LDA) scores greater than 2.0.

The relative abundance of intestinal flora at the phylum level was shown in Figure 3A. The ratio of *Firmicutes/Bacteroidetes* (F/B) in the B13 group was higher than that in the control group. In the top 11 genera (the relative abundance > 0.01), the relative abundance of *Muribaculaceae*, *Lachnospiraceae_NK4A136_group* in B13 group was a certain increase, but the difference is not significant, and that of *Alloprevotella* and *Bacteroides* in B13 group was significantly reduced (*p* < 0.05) (Figure 3B).

Further PCoA analysis (Figure 3C) showed that the two groups were not completely separated, but Adonis analysis showed a difference (*p* = 0.029). It suggested that under the conditions of this study, there were differences in the structure of colonic flora between the two groups. Although *E. faecium B13* did not change Alpha diversity, it indeed affected the composition. LEfSe analysis with an LDA threshold of two showed that there were 13 differential biomarkers (*p* < 0.05) at the genus level between the two groups (Figure 3D), and among them, *Alloprevotella*, *Bacteroides*, *Faecalibacterium*, *Butyricimonas* were downregulated significantly, while *Faecalibaculum*, *Candidatus_Stoquefichus* and *Parasutterella* were significantly upregulated. Many studies showed that *Alloprevotella, Bacteroides*, *Faecalibacterium*, and *Butyricimonas* were negatively correlated with obesity (Li et al. 2020; Chen et al. 2020) and *Parasutterella* is positively correlated with obesity (Sun et al. 2024). Moreover, *Faecalibaculum* and *Candidatus_Stoquefichus* belonged to *Firmicutes* (Liu, Qin, et al. 2021), so the relative abundance of *Firmicutes* rose in the B13 group.

Comparing the composition of microbiota in the colon and ileum, it can be found that there are significant differences, and there are more uncultured microbes in the colon. Based on 16S rRNA sequencing data, Phylogenetic Investigation of Communities by Reconstruction of Unobserved States (PICRUSt) analysis was used to predict the function of intestinal flora. There were 26 differential KEGG pathways in the two groups (*p* < 0.05), among which PPAR signaling pathway, adipocytokine signaling pathway and arachidonic acid metabolism were related to lipid metabolism (Table S3).

### 3.5 Analysis of Metabolites in Colon

**FIGURE 4:**
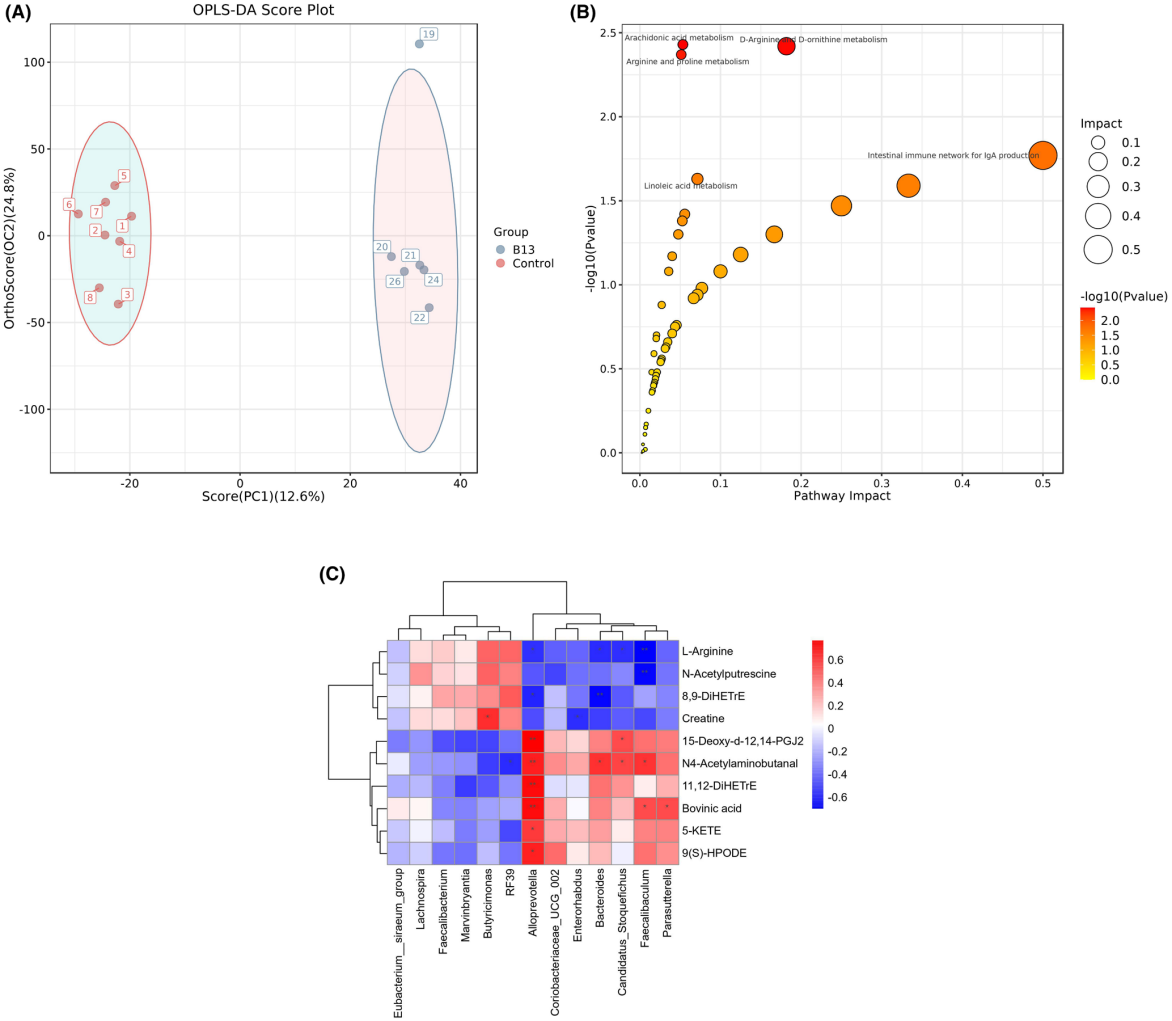
Metabolite analysis of colonic contents. (A) OPLS‐DA score of colonic metabolite profiles. (B) KEGG functional enrichment analysis. Each point represents a metabolic pathway. The horizontal axis represents the impact values enriched in different metabolic pathways; the vertical axis represents −log10 (*p*‐value) tested by hypergeometric distribution. The *p*‐value is smaller, and the impact of detected differential metabolites on this pathway is more significant; the *p*‐value is smaller, and the color is darker. The impact value represents the degree of contribution of metabolites detected under this pathway; the impact value is larger; the dot is bigger. (C) Correlation analysis between significantly different microbes and major metabolites in the colon. The color indicates Spearman's correlation coefficient, and significant correlations are noted by adjusted *p*, *p < 0.05, **p < 0.01.

Following the PICRUSt analysis, a non‐targeted metabolome analysis was further performed on colonic content. Based on OPLS‐DA score (Figure 4A), 53 biomarkers were identified using Student's *t*‐test and non‐parametric test (VIP > 1, *p* < 0.05), including lipids and lipid‐like molecules (18), organic acids and their derivatives (10), nucleosides, nucleotides and analogs (3), organic nitrogen compounds (8), organic oxygen compounds (2), alkaloids and their derivatives (1), benzenes (3), phenylacetones and polyketides (3), unclassified metabolites (5) (Figure S2). A total of 13 biomarkers were upregulated and 40 downregulated, compared with the control group.

KEGG functional enrichment analysis was further performed on the differential metabolites. Results showed there were significant differences in four metabolic pathways between the two groups, including the arachidonic acid metabolic pathway, arginine and ornithine metabolic pathway, arginine and proline metabolic pathway, and linoleic acid metabolic pathway (Figure 4B). The arachidonic acid metabolic pathway and linoleic acid metabolic pathway were related to obesity (Wang et al. 2021; Wang and Wang 2023). Most metabolites in the two pathways, except for 8,9‐DiHETrE, were downregulated, including 11,12‐DiHETrE, 15‐Deoxy‐d‐12,14‐PGJ2, and Bovinic acid, all of which were negatively correlated with obesity (Cai et al. 2022; Xu, Zhu, et al. 2022). In contrast, in the arginine and ornithine metabolic pathway and arginine and proline metabolic pathway, the content of most metabolites increased, especially L‐arginine.

### 3.6 Correlation Analysis Between Colonic Flora and Differential Metabolites

The relationship between differential metabolites in 4 significantly different pathways and microbial biomarkers was analyzed with Spearman analysis (Figure 4C).

*Alloprevotella* is the main different genus and significantly impacts these differential metabolites. It was positively correlated with lipid metabolites and negatively correlated with amino acid metabolites. This result once again proves that *Alloprevotella* can prevent and treat obesity. The effect of *Bacteroides* on these substances was consistent with that of *Alloprevotella*.

### 3.7 Colonic Microbiota on 11th Day After Stopping Feeding B13

**FIGURE 5:**
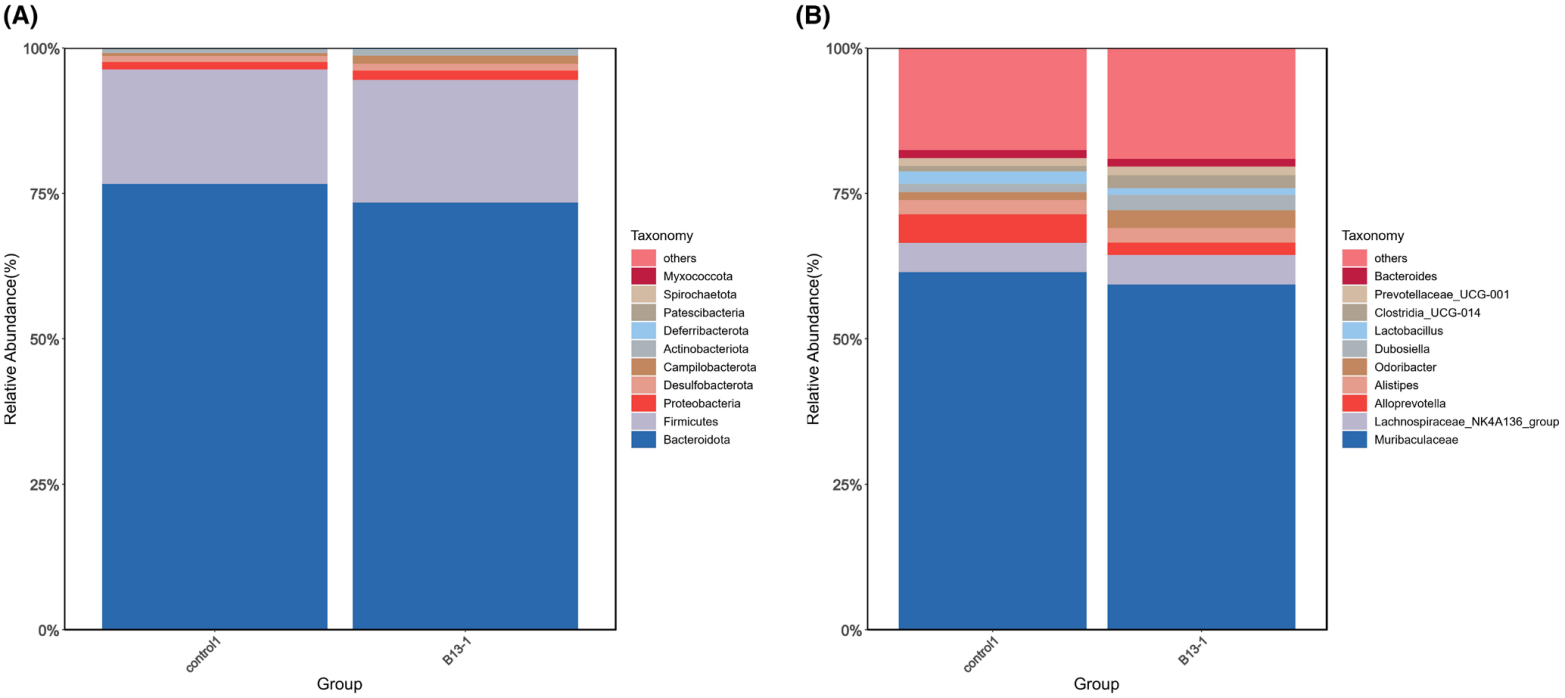
Community composition in colon at the phylum and genus levels on day 38. (A) Phylum‐level species distribution bar chart, showing only the top 10 species; (B) Genus‐level species distribution bar chart, showing only the top 10 species. Control = control group; B13‐1 = treatment group maintained on regular feed for 10 days following stopping B13 oral gavage.

In order to determine the sustained impact of B13 on colonic microbiota, colonic microbiota was analyzed on the 11th day after stopping feeding B13. The microbial composition at the phylum and genus levels is shown in Figure 5A,B. At the phylum level, the ratio of F/B remains higher than the control group, but the value decreased on the 11th day after stopping feeding compared with that on the 29th day during feeding. At the genus level, the relative abundance of all significantly altered genera was no longer significantly different from that of the control group, except *Alloprevotella* and *RF39*.

*Alloprevotella* was still low in the B13 group (*p* < 0.05), suggesting that *Alloprevotella*'s abundance might not be exclusively determined by the abundance of B13, but rather be predominantly regulated by the gut microenvironment. Notably, *Parasutterella* showed no difference between the two groups, indicating that it would not further cause pathological changes. The relative abundance of *Muribaculaceae* decreased (*p* > 0.05), Although the change was not significant, the trend was completely consistent with whether B13 was added, which indicated that the *Muribaculaceae* might be directly affected by B13.

## 4 Discussion

Understanding the effects of feeding *E. faecium* on animal health, growth performance, and gut microbiota is important for evaluating its probiotic properties. In this study, although no significant differences were observed in final body weight, ADG, ADFI, and F/R, mice in the B13 group showed higher body weight than the control group during the feeding phase. While the difference was not statistically significant, this trend was consistent with our previous findings in piglets. Since growth performance did not change significantly in this experiment, future studies will consider increasing the sample size or the dosage of B13 to further validate these results.

In the present study, changes in the ileal microbiota were minimal, and only *Allobaculum* among the top 30 genera showed a significant change. Many studies have shown that it can produce short‐chain fatty acids (SCFA), such as lactic acid and butyric acid (Janssen et al. 2018; Li et al. 2019), is negatively correlated with blood lipid levels (Mu et al. 2020; Wang et al. 2023; Hou et al. 2023), and can reduce the accumulation of δ‐VB and the occurrence of obesity (Mossad et al. 2021; Bustos et al. 2018; Liu, Owens, et al. 2021). All the studies analyzed the effect of *Allobaculum* in the colon; therefore, further analysis is needed on the function of the bacterium in the ileum.

However, B13 had a significantimpact on colonic microbiota. There were 5 significantly different microbes in the top 30 genera, including *Alloprevotella* and *Bacteroides*. Compared with the control group, *Alloprevotella*, *Bacteroides*, *Faecalibacterium*, *Butyricimonas* and *RF39*, significantly decreased in group B13. Many studies (Sun et al. 2021; Zhou et al. 2018) have shown that these strains can produce SCFAs, such as butyric acid and propionic acid, etc. SCFAs may stimulate epithelial cell proliferation and play an important role in improving gut barrier integrity, with the potential to suppress LPS release and anti‐obesity activity. Thus, these strains are often negatively correlated with obesity. In particular, *Alloprevotella and Bacteroides* can regulate lipid metabolism by modulating the PPAR signaling pathway. Furthermore, many studies have proved that *Parasutterella* promotes obesity and is closely related to irritable bowel syndrome (IBS) and chronic intestinal inflammation (Butler et al. 2023; Rodziewicz et al. 2024) but anti‐inflammatory bacteria, like *Enterorhabdus* (Cheng et al. 2023), and anti‐obesity bacteria, like *Coriobacteriaceae_UCG_002* and *Faecalibaculum* (Jiao et al. 2023; Mu et al. 2021; Kawano et al. 2022), are also upregulated in the B13 group. The upregulated *Parasutterella* in the B13 group may produce side effects; therefore, microbiomes were detected 10 days after the cessation of gavage. A significant difference in the abundance of *Alloprevotella* and *RF39* was still observed between the two groups, while no differences were observed in all other altered bacterial genera. The findings indicated that stopping B13 gavage does not lead to sustained inflammation.

The Firmicutes/Bacteroidetes (F/B) ratio is an important parameter and high values of F/B were considered an important indicator of obesity (Abenavoli et al. 2019; Palmas et al. 2021). Our study showed that feeding B13 for 28 days caused the ratio of F/B in the ileum to be slightly down; the reason needs further analysis. However, the ratio of F/B in the colon was up, whether it was the 28‐day feeding period or 10 days after stopping feeding, which may be related to the significant downregulation of *Alloprevotell*. The ratio of F/B was inconsistent in different intestinal parts in this study. The result is the same as that of Zhou et al. (2021).

Many studies have shown that *E. faecium* plays a role in reducing lipid levels such as TG, TC, and LDL‐C in animal serum. *E. faecium 132* could significantly reduce serum TG and LDL‐C levels in SD male rats (Xu, Zou, et al. 2022), with similar results reported for *E. faecium GEFA01* (Yang et al. 2021). This study found that by feeding *E. faecium B13* for 4 weeks, the level of TG in the serum of mice was significantly reduced, and the level of TC was also decreased. Serum TC is the main raw material for the synthesis of cell membranes, steroid hormones, and bile acids in animal bodies, and serum TG is mainly used for energy metabolism (Rodriguez et al. 2020). The decrease in serum TG and TC levels may be related to the upregulation of *Allobaculum* in the ileum.

Colonic metabolome analysis revealed that four metabolic pathways significantly changed. In the arachidonic acid metabolic pathway and linoleic acid metabolic pathway, most differential metabolites were reduced, such as 15‐Deoxy‐d‐12,14‐PGJ2, 11,12‐DiHETrE (Shen et al. 2022), and Bovinic acid. 15‐Deoxy‐d‐12,14‐PGJ2 is a form of prostaglandin D_2_ (PGD), and low concentrations of 15‐Deoxy‐d‐12,14‐PGJ2 can promote lipid accumulation by modulating the PPARγ signaling pathway. Like 15‐Deoxy‐Δ12,14‐PGJ2, 11,12‐DiHETrE and Bovinic acid can promote lipid accumulation by modulating the PPARγ signaling pathway too (Li et al. 2023; Kim et al. 2013) PPAR‐γ is a key transcription factor for adipocyte differentiation in adipose tissue, and can activate adipocyte formation and promote adipocyte differentiation (Wang et al. 2020; McReynolds et al. 2023). Correlation analysis showed that 11,12‐DiHETrE and 15d‐PGJ2 were positively correlated with *Alloprevotella*, *Bacteroides* and all these intestinal florae upregulated in the B13 group. Studies have shown that many arachidonic acid (AA) metabolites are significantly positively correlated with the abundance of *Alloprevotilla* (Zhang et al. 2022). The accumulation of AA metabolites may further alter the intestinal microenvironment, affecting the survival or proliferation of *Alloprevototella* in a potentially bidirectional relationship. Notably, 8,9‐DiHETrE increased in the B13 group and its effect is without deep reports.

In the arginine, ornithine and proline metabolic pathway, major metabolites, such as L‐arginine, significantly increased. Most of these differential metabolites were significantly correlated with *Alloprevotella*, which was consistent with the results of microbial community analysis. In addition, the previous study (Xiao et al. 2024) had shown that B13 can degrade arginine, which may be the reason for the corresponding changes in amino acid metabolism pathways. Further research is needed to investigate the effect of B13 on arginine in the intestine.

## 5 Conclusion

Overall, our findings suggest a potential role for *E. faecium B13* in improving growth performance, possibly mediated through the modulation of the gut microbiota and metabolites. In particular, *Alloprevotella* and arginine are proposed as potential key targets regulated by B13. Spearman's correlation analysis revealed that *Alloprevotella* is related to the fatty acid belonging to the arachidonic acid metabolic and linoleic acid metabolic pathways. Questions regarding the impact of arginine on gut microbiota and intestinal health during this process, and the dose and feeding time effects of B13 remain open and are the subject of further studies.

## Author Contributions

**Fan Luo:** conceptualization (equal), data curation (equal), project administration (equal), writing – review and editing (equal). **Jiajun Wu:** investigation (equal), resources (equal), writing – original draft (equal). **Shumin Wang:** methodology (supporting). **Ming Zhang:** methodology (supporting). **Yixuan Song:** validation (supporting). **Yaqiu Lin:** funding acquisition (equal), supervision (supporting).

## Ethics Statement

The animal study was reviewed by the Animal Ethics Committee of Southwest University for Nationalities (Chengdu, China) (license number: SMU20201206).

## Conflicts of Interest

The authors declare no conflicts of interest.

## Supporting information


**Table S1:** Effect of *E. faecium B13* on the growth performance in mice.
**Table S2:** Effect of *E. faecium B13* on organ indices in mice.
**Table S3:** Effect of *E. faecium B13* on differential metabolic pathways in the colon of mice with PICRUSt analysis. Control group, basal diet; B13 group, basal diet supplemented with 1 × 10^8^ CFU/mL *E. faecium* B13.
**Figure S1:** Heatmap of Spearman correlation coefficients between different microbes and 53 metabolites. R values are shown in different colors in the graph, in which red indicates positive correlation and blue indicates negative correlation. *, **, and *** represent *p* < 0.05, *p* < 0.01 and *p* < 0.001.

## Data Availability

The raw sequences generated for this study can be found in the NCBI Short Read Archive under Bio Project PRJNA1149902. Metabolome data can be found in the OMIX database (OMIX007155).
