# Supplementary material for: Enterococcus faecium B13 Affects Mice Growth by Regulating Gut Microbiota and Metabolites
Source: Food Sci Nutr. 2025 Nov 28;13(12):e71192. doi: 10.1002/fsn3.71192 (PMC12661549; doi:10.1002/fsn3.71192)
Supplement: Supplementary file 1 — Table S1: Effect of E. faecium B13 on the growth performance in mice. Table S2: Effect of E. faecium B13 on organ indices in mice. Table S3: Effect of E. faecium B13 on differential metabolic pathways in the colon of mice with PICRUSt analysis. Control group, basal diet; B13 group, basal diet supplemented with 1 × 10^8^ CFU/mL E. faecium B13. Figure S1: Heatmap of Spearman correlation coefficients between different microbes and 53 metabolites. R values are shown in different colors in the graph, in which red indicates positive correlation and blue indicates negative correlation. *, **, and *** represent p < 0.05, p < 0.01 and p < 0.001. [file FSN3-13-e71192-s001.docx]

**Table S1.** Effect of *E. faecium B13* on the growth performance in mice on day 28

| **Items** | **Control Group** | **B13 Group** | ***p*-value** |
| --- | --- | --- | --- |
| Initial weight/(g) | 19.59±0.95 | 20.08±0.81 | 0.656 |
| Final weight/(g) | 35.94±1.57 | 37.94±1.95 | 0.652 |
| Average daily weight 0.58±0.05 | | 0.64±0.07 | 0.168 |
| Average daily feed 6.03±0.12 | | 6.73±0.09 | 0.184 |
| Feed-to-weight ratio 10.33±1.44 | | 10.55±1.72 | 0.763 |
| **Table S2.** Effect of *E. faecium B13* on organ indices in mice on day 28 | | | |
| **Items** | **Control Group** | **B13 Group** | ***p*-value** |
| Cardiac index | 6.15±1.22 | 5.57±0.62 | 0.308 |
| Liver index | 49.17±3.27 | 48.68±5.18 | 0.200 |
| Spleen index | 3.27±0.78 | 3.23±0.76 | 0.088 |
| Kidney index | 19.85±1.78 | 18.24±3.79 | 0.071 |

gain/(g/d) intake/(g/d) (F/R)

**Table S3.** Effect of *E. faecium B13* on differential metabolic pathways in the colon of mice with PICRUSt analysis

| **Index** | **Control Group** | **B13 Group** | ***P*-valve** |
| --- | --- | --- | --- |
| Adipocytokine signaling pathway | 68631.97±9664.69 | 51704.25±7754.5^**^ | <0.01 |
| Apoptosis | 42118.08±7159.56 | 28969.23±6345.71^**^ | <0.01 |
| Arachidonic acid metabolism | 21702.62±3494.55 | 17386.53±2236.51^*^ | 0.02 |
| Biosynthesis of siderophore group |  |  | 0.01 |

nonribosomal peptides Carbohydrate digestion and absorption

Ferroptosis Folate biosynthesis

Glycosphingolipid biosynthesis -

<0.01

<0.01

| 19567.93±2986.34 | 14032.17±3632.58^*^ |
| --- | --- |
| 10895.08±4461.13 | 4135.86±2323.47^**^ |
| 88729.69±11797.27 | 65671.2±11251.06^**^ |
| 363220.61±63233.23 | 297415.05±36035.97^*^ |
| 76887.63±14425.63 | 54745.91±12018.89^**^ |

0.03

<0.01

biosynthesis

| ganglio series |  | | |
| --- | --- | --- | --- |
| Huntingtons disease | 43106.63±9397.42 | 33240.6±5823.6^*^ | 0.03 |
| Isoquinoline alkaloid biosynthesis | 50926.29±7914.83 | 43119.45±5364.97^*^ | 0.05 |
| Lipoic acid metabolism | 40961.63±9267.77 | 27405.93±4304.71^**^ | <0.01 |
| Lipopolysaccharide biosynthesis | 324968.26±48644.67 | 248881.38±26167.73^**^ | <0.01 |
| Nitrotoluene degradation | 31133.21±1895.85 | 36501.34±3392.55^**^ | <0.01 |
| Other glycan degradation | 329464.97±56846.09 | 258424.33±43617.77^*^ | 0.02 |
| PPAR signaling pathway | 105793.31±16516.58 | 87248.19±9750.72^*^ | 0.02 |
| Pancreatic secretion | 9260.46±3454.35 | 3710.52±2086.08^**^ | <0.01 |
| Peroxisome | 126160.33±22816.28 | 101027.95±15003.2^*^ | 0.03 |
| Pertussis | 36633.96±5063.41 | 28695.64±5448.9^*^ | 0.02 |
| Protein digestion and absorption | 32590.36±7431.51 | 18585.77±6036.82^**^ | <0.01 |
| Protein processing in endoplasmic | 68173.6±9291.03 | 58131.49±5770.53^*^ | 0.03 |
| reticulum |  |  |  |
| Renin-angiotensin system | 3705.03±1660.37 | 1601.09±425.13^**^ | <0.01 |
| Salivary secretion | 9237.56±3465.91 | 3687.27±2077.56^**^ | <0.01 |
| Thyroid hormone synthesis | 12028.91±4156.07 | 6809.61±1888.81^*^ | 0.01 |
| Ubiquinone and other terpenoid- |  |  | <0.01 |
| 158549.32±25504.76 | | 123709.44±11576.04^**^ | |
| quinone biosynthesis  Various types of N-glycan |  |  | <0.01 |
| 77392.31±14483.43 | | 55417.71±11492.62^**^ |  |
| Zeatin biosynthesis 50733.74±7174.98 | | 41975.33±4666.74^*^ | 0.02 |

* Indicates significant difference between the means of the Control group and the B13 group (*p*<0.05), ^**^ Indicates extremely significant difference between the means of the Control group and the B13 group (*p*<0.01).


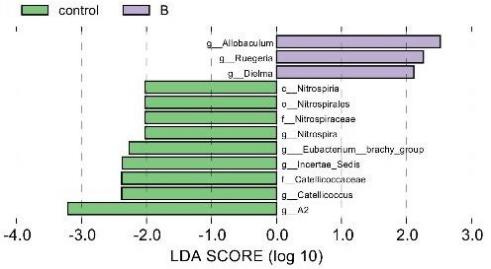


**Figure. S1.** LEfSe linear discriminant analysis of the ileum microflora.


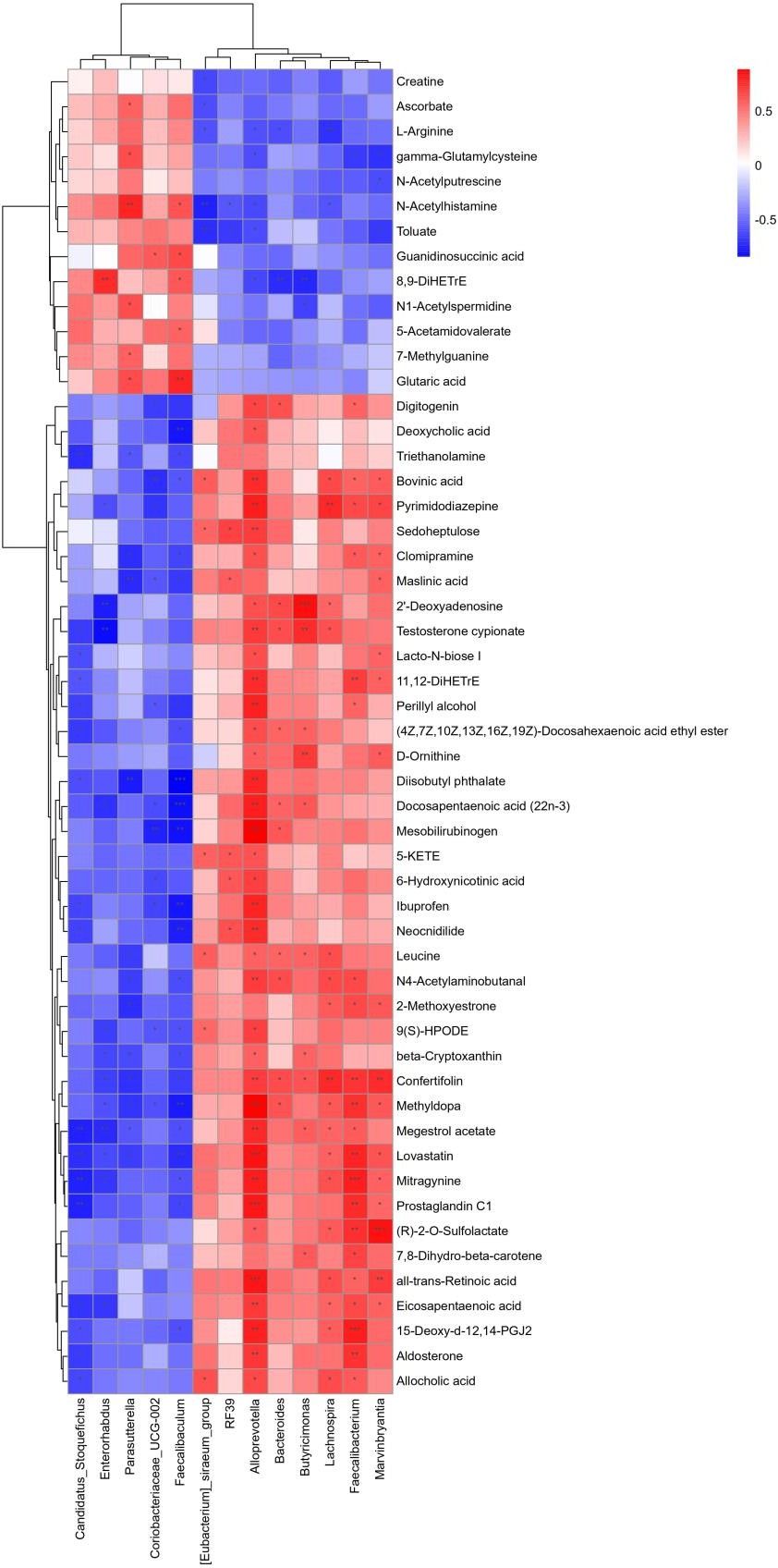


**Figure S2.** Heatmap of Spearman correlation coefficients between different microbes and 53 metabolites.

R values are shown in different colors in the graph, in which red indicates positive correlation and blue indicates negative correlation. *, **, and *** represent *p*<0.05, *p*<0.01, *p*<0.001.
